# 

**Published:** 1875-06-01

**Authors:** 


### CONTENTS:

*ORIGINAL COMMUNICATIONS:*

Report of Three Cases of Acute Cerebral Softening. By D. A. K. Steele, M. D. ........ 297

*TRANSLATIONS:*

Clinical Thermometry. By Dr. B. Kraus. Condensed and Translated by Dr. H. Gradle, .... 300

*EDITORIAL DEPARTMENT:*

"One of the Lost Arts," ........ 305

*SOCIETY REPORTS:*

Chicago Society of Physicians and Surgeons. Regular Meeting, May 10th, 1875. Reported by E. Warren Sawyer, M.D. ........ 308

Regular Meeting, May 24, 1875. Reported by E. Warren Sawyer, M.D. ........ 313

Illinois State Medical Society, ........ 315

*GLEANINGS FROM OUR EXCHANGES:*

Bastian on the Germ Theory of Disease, ........ 320

Zeissl's Mode of Introducing Liquids into the Male Bladder without the Use of the Catheter. By A. Rose, M.D. ........ 322

